# Crystal structure of ethyl 2-{4-[(5-chloro-1-benzo­furan-2-yl)meth­yl]-3-methyl-6-oxo-1,6-di­hydro­pyridazin-1-yl}acetate

**DOI:** 10.1107/S2056989015006301

**Published:** 2015-04-09

**Authors:** Youness Boukharsa, Lahcen El Ammari, Jamal Taoufik, Mohamed Saadi, M’hammed Ansar

**Affiliations:** a Laboratory of Medicinal Chemistry, Faculty of Medicine and Pharmacy, BP 6203, Rabat Institute, University Mohammed V, Rabat, Morocco; b Laboratoire de Chimie du Solide Appliquée, Faculté des Sciences, Université Mohammed V, Avenue Ibn Battouta, BP. 1014, Rabat, Morocco

**Keywords:** crystal structure, pyridazinone derivative, hydrogen bonding

## Abstract

In the title compound, C_18_H_17_ClN_2_O_4_, the dihedral angle between the benzofuran ring system [maximum deviation 0.014 (2) Å] and the oxopyradizine ring is 73.33 (8)°. The structure is characterized by disorder of the ethyl group, which is split into two parts, with a major component of 0.57 (3), and the acetate carbonyl O atom, which is statistically disordered. In the crystal, the molecules are linked by C—H⋯O inter­actions, forming a three-dimensional network.

## Related literature   

For pharmacological activities of pyridazinones, *e.g*. anti­microbial, see: Boukharsa *et al.* (2014 ▸); Nagle *et al.* (2014 ▸); El-Hashash *et al.* (2014 ▸); Tiryaki *et al.* (2013 ▸); Csókás *et al.* (2013 ▸); Asif *et al.* (2014 ▸); Garkani-Nejad & Poshteh-Shirani (2013 ▸). For biological activities of pyridazinone derivatives and their applications, *e.g*. as insecticides and herbicides, see: Cao *et al.* (2003 ▸); Jamet & Piedallu (1975 ▸). For pyridazin-3(2*H*)-one derivatives, see: Taoufik *et al.* (1984 ▸); Benchat *et al.* (1998 ▸); Abourichaa *et al.* (2003 ▸).
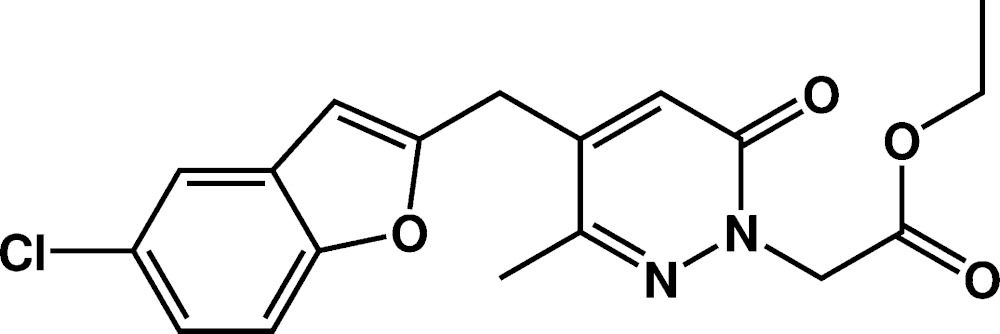


## Experimental   

### Crystal data  

C_18_H_17_ClN_2_O_4_
*M* *_r_* = 360.79
Orthorhombic, 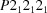

*a* = 7.9792 (2) Å
*b* = 8.7460 (2) Å
*c* = 25.2064 (6) Å
*V* = 1759.06 (7) Å^3^
*Z* = 4
Mo *K*α radiation
μ = 0.24 mm^−1^
*T* = 296 K
0.37 × 0.34 × 0.29 mm

### Data collection  

Bruker APEXII CCD diffractometer
Absorption correction: multi-scan (*SADABS*; Bruker, 2009 ▸) *T*_min_ = 0.589, *T*_max_ = 0.746
12689 measured reflections
4925 independent reflections
3350 reflections with *I* > 2σ(*I*)
*R*_int_ = 0.031

### Refinement  

*R*[*F* ^2^ > 2σ(*F* ^2^)] = 0.044
*wR*(*F* ^2^) = 0.114
*S* = 1.02
4925 reflections
255 parameters
H-atom parameters constrained
Δρ_max_ = 0.25 e Å^−3^
Δρ_min_ = −0.34 e Å^−3^
Absolute structure: Flack & Bernardinelli (2000 ▸), 2104 Friedel pairs
Absolute structure parameter: 0.02 (7)

### 

Data collection: *APEX2* (Bruker, 2009 ▸); cell refinement: *SAINT* (Bruker, 2009 ▸); data reduction: *SAINT*; program(s) used to solve structure: *SHELXS97* (Sheldrick, 2008 ▸); program(s) used to refine structure: *SHELXL97* (Sheldrick, 2008 ▸); molecular graphics: *ORTEP-3 for Windows* (Farrugia, 2012 ▸) and *PLATON* (Spek, 2009 ▸); software used to prepare material for publication: *publCIF* (Westrip, 2010 ▸).

## Supplementary Material

Crystal structure: contains datablock(s) I. DOI: 10.1107/S2056989015006301/tk5363sup1.cif

Structure factors: contains datablock(s) I. DOI: 10.1107/S2056989015006301/tk5363Isup2.hkl

Supporting information file. DOI: 10.1107/S2056989015006301/tk5363Isup3.cml

**Fig. 1.**
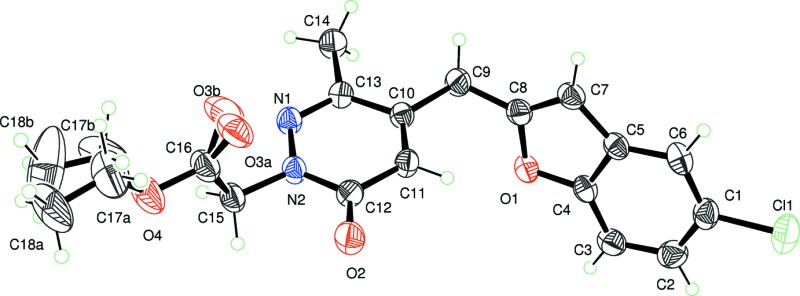
Molecular structure of the title compound with the atom-labelling scheme. Displacement ellipsoids are drawn at the 50% probability level. H atoms are represented as small circles.

. DOI: 10.1107/S2056989015006301/tk5363fig1.tif
Mol­ecular structure of the title compound with the atom-labelling scheme. Displacement ellipsoids are drawn at the 50% probability level. H atoms are represented as small circles.

**Fig. 2.**
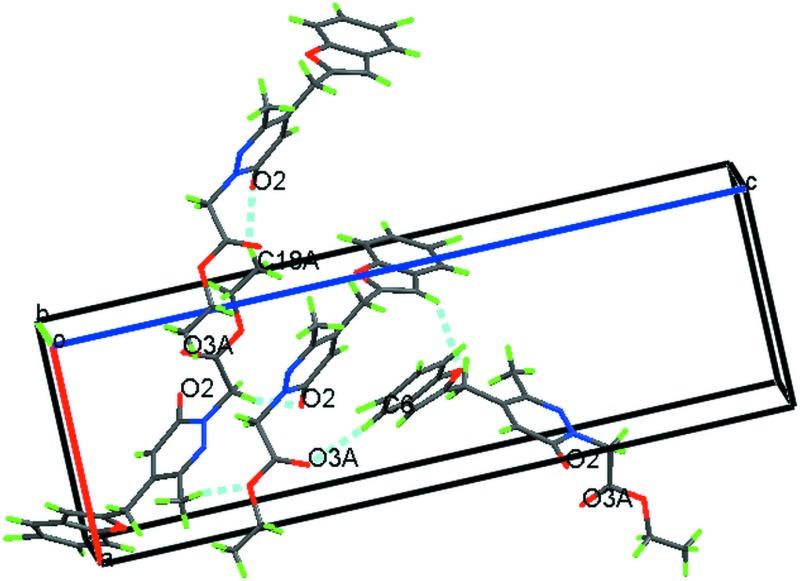
Crystal packing in the structure of the title compound, showing molecules linked by C—H···O hydrogen bonds (dashed lines).

. DOI: 10.1107/S2056989015006301/tk5363fig2.tif
Crystal packing in the structure of the title compound, showing mol­ecules linked by C—H⋯O hydrogen bonds (dashed lines).

CCDC reference: 1056731

Additional supporting information:  crystallographic information; 3D view; checkCIF report

## Figures and Tables

**Table 1:** Hydrogen-bond geometry (, )

*D*H*A*	*D*H	H*A*	*D* *A*	*D*H*A*
C6H6O3*B* ^i^	0.93	2.37	3.291(19)	170
C15H15*B*O2^ii^	0.97	2.34	3.278(3)	161
C18*A*H18*A*O2^iii^	0.96	2.41	3.310(10)	156

Symmetry codes: (i) 
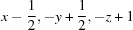
; (ii) 
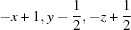
; (iii) 
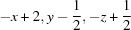
.

## supplementary crystallographic information

### S1. Comment

During recent years, pyridazinones have been a subject of numerous recent studies (Boukharsa *et al.*, 2014) owing to their wide spectrum of pharmacological activities such as antimicrobial (Nagle *et al.*, 2014), anti-fungal (El-Hashash *et al.*, 2014), analgesic & anti-inflammatory (Tiryaki *et al.*, 2013), anticancer (Csókás *et al.*, 2013), anti-tubercular (Asif *et al.*, 2014) and anti-hypertensive activities (Garkani-Nejad & Poshteh-Shirani, 2013). It has also been reported that pyridazinone derivative have remarkable insecticidal (Cao *et al.*, 2003) and herbicidal activities (Jamet & Piedallu, 1975). In continuation of this line of research (Taoufik *et al.*, 1984; Benchat *et al.*, 1998; Abourichaa *et al.*, 2003), we have developed a new pyridazin-3(2*H*)-one derivative. It will be subjected to further pharmacological investigations, especially tests of anticancer activity. Compound (I) is stable at room temperature, and its structure has been determined by NMR (^1^H and ^13^ C). In this paper we wish to report the crystal structure determination of the title compound possessing the biologically active pyridazinone ring.

The molecule of the title compound is build up from 5-chlorobenzofuran-2-yl linked, *via* –CH_2_– group, to six-membered heterocyclic ring which is related to acetate group as shown in Fig. 1. The benzofuran system is virtually planar with the largest deviation from the mean plane being -0.014 (2) Å at C4, and makes dihedral angle of 73.33 (8)° with the mean plane through the oxopyridazin (C10–C13,N1,N2) ring. Non classical C—H···O hydrogen bonds link the molecules into a three-dimensional network.

### S2. Experimental

To a solution of 5-((5-chlorobenzofuran-2-yl)methyl)-6-methylpyridazin-3(2*H*)-one (0.5 g, 1.82 mmol) dissolved in tetrahydrofuran (15 ml) was added ethyl 2-bromoacetate (0.30 ml, 2.73 mmol), potassium carbonate (0.5 g, 3.64 mmol) and a catalytic amount of tetra-n-butylammonium bromide (0.05 g, 0.15 mmol). The mixture was stirred at room temperature for 6 h, and monitored by thin layer chromatography. The compound was removed by filtration and the filtrate concentrated under vacuum. The solid obtained was crystallized from ethanol to afford colourless crystals (Yield = 77%; M.pt = 136.9 °C).

### S3. Refinement

The H atoms were located in a difference map and treated as riding with C—H = 0.93 Å (aromatic), C—H = 0.97 Å (methylene) and C—H = 0.96 Å (methyl), and with *U*_iso_(H) = 1.2 *U*_eq_ (aromatic and methylene) and *U*_iso_(H) = 1.5 *U*_eq_ for methyl. This structure is characterized by a partial disorder at the acetate group, with the ethyl group split into two parts. The major component had a site occupancy factor = 0.57 (3). The carbonyl-O3 was statistically disordered. Owing to poor agreement, the (0 0 2) reflection was omitted from the final cycles of refinement.

### Crystal data

**Table:**

C_18_H_17_ClN_2_O_4_	*F*(000) = 752
*M**_r_* = 360.79	*D*_x_ = 1.362 Mg m^−^^3^
Orthorhombic, *P*2_1_2_1_2_1_	Mo *K*α radiation, λ = 0.71073 Å
Hall symbol: p 2ac 2ab	Cell parameters from 4925 reflections
*a* = 7.9792 (2) Å	θ = 2.5–29.6°
*b* = 8.7460 (2) Å	µ = 0.24 mm^−^^1^
*c* = 25.2064 (6) Å	*T* = 296 K
*V* = 1759.06 (7) Å^3^	Block, colourless
*Z* = 4	0.37 × 0.34 × 0.29 mm

### Data collection

**Table:**

Bruker APEXII CCD diffractometer	4925 independent reflections
Radiation source: fine-focus sealed tube	3350 reflections with *I* > 2σ(*I*)
Graphite monochromator	*R*_int_ = 0.031
ω and φ scans	θ_max_ = 29.6°, θ_min_ = 2.5°
Absorption correction: multi-scan (*SADABS*; Bruker, 2009)	*h* = −11→6
*T*_min_ = 0.589, *T*_max_ = 0.746	*k* = −12→11
12689 measured reflections	*l* = −35→34

### Refinement

**Table:**

Refinement on *F*^2^	Secondary atom site location: difference Fourier map
Least-squares matrix: full	Hydrogen site location: difference Fourier map
*R*[*F*^2^ > 2σ(*F*^2^)] = 0.044	H-atom parameters constrained
*wR*(*F*^2^) = 0.114	*w* = 1/[σ^2^(*F*_o_^2^) + (0.0511*P*)^2^ + 0.0996*P*] where *P* = (*F*_o_^2^ + 2*F*_c_^2^)/3
*S* = 1.02	(Δ/σ)_max_ = 0.043
4925 reflections	Δρ_max_ = 0.25 e Å^−^^3^
255 parameters	Δρ_min_ = −0.34 e Å^−^^3^
0 restraints	Absolute structure: Flack & Bernardinelli (2000), 2104 Friedel pairs
Primary atom site location: structure-invariant direct methods	Absolute structure parameter: 0.02 (7)

### Special details

**Table:**

Geometry. All e.s.d.'s (except the e.s.d. in the dihedral angle between two l.s. planes) are estimated using the full covariance matrix. The cell e.s.d.'s are taken into account individually in the estimation of e.s.d.'s in distances, angles and torsion angles; correlations between e.s.d.'s in cell parameters are only used when they are defined by crystal symmetry. An approximate (isotropic) treatment of cell e.s.d.'s is used for estimating e.s.d.'s involving l.s. planes.
Refinement. Refinement of *F*^2^ against all reflections. The weighted *R*-factor *wR* and goodness of fit *S* are based on *F*^2^, conventional *R*-factors *R* are based on *F*, with *F* set to zero for negative *F*^2^. The threshold expression of *F*^2^ > σ(*F*^2^) is used only for calculating *R*-factors(gt) *etc*. and is not relevant to the choice of reflections for refinement. *R*-factors based on *F*^2^ are statistically about twice as large as those based on *F*, and *R*- factors based on all data will be even larger.

### Fractional atomic coordinates and isotropic or equivalent isotropic displacement parameters (Å^2^)

**Table:**

	*x*	*y*	*z*	*U*_iso_*/*U*_eq_	Occ. (<1)
C1	0.0331 (3)	0.7077 (3)	0.55647 (8)	0.0497 (5)	
C2	−0.0495 (3)	0.7389 (3)	0.50905 (9)	0.0551 (6)	
H2	−0.1006	0.8335	0.5042	0.066*	
C3	−0.0561 (3)	0.6314 (3)	0.46924 (8)	0.0500 (5)	
H3	−0.1101	0.6507	0.4372	0.060*	
C4	0.0215 (2)	0.4935 (2)	0.47935 (7)	0.0408 (4)	
C5	0.1023 (2)	0.4581 (2)	0.52669 (7)	0.0401 (5)	
C6	0.1087 (3)	0.5691 (3)	0.56674 (8)	0.0493 (6)	
H6	0.1618	0.5500	0.5989	0.059*	
C7	0.1597 (3)	0.3032 (3)	0.52045 (8)	0.0448 (5)	
H7	0.2188	0.2460	0.5453	0.054*	
C8	0.1118 (3)	0.2568 (3)	0.47190 (8)	0.0431 (5)	
C9	0.1316 (3)	0.1120 (3)	0.44154 (8)	0.0472 (5)	
H9A	0.0220	0.0785	0.4297	0.057*	
H9B	0.1755	0.0342	0.4652	0.057*	
C10	0.2463 (2)	0.1236 (2)	0.39358 (7)	0.0368 (4)	
C11	0.3432 (2)	0.2461 (2)	0.38377 (7)	0.0414 (4)	
H11	0.3383	0.3299	0.4064	0.050*	
C12	0.4547 (3)	0.2499 (2)	0.33859 (8)	0.0409 (4)	
C13	0.2541 (2)	−0.0037 (3)	0.35783 (7)	0.0384 (4)	
C14	0.1523 (3)	−0.1455 (3)	0.36636 (9)	0.0514 (5)	
H14A	0.1751	−0.2173	0.3385	0.077*	
H14B	0.0353	−0.1198	0.3662	0.077*	
H14C	0.1812	−0.1902	0.3999	0.077*	
C15	0.5492 (3)	0.1158 (3)	0.25914 (8)	0.0460 (5)	
H15A	0.5611	0.2173	0.2441	0.055*	
H15B	0.4950	0.0513	0.2329	0.055*	
C16	0.7196 (3)	0.0523 (3)	0.27179 (9)	0.0570 (6)	
C17A	0.9885 (15)	0.004 (2)	0.2313 (4)	0.090 (4)	0.57 (3)
H17A	1.0439	0.0692	0.2571	0.108*	0.57 (3)
H17B	0.9939	−0.0998	0.2446	0.108*	0.57 (3)
C18A	1.0731 (14)	0.0109 (18)	0.1866 (4)	0.115 (5)	0.57 (3)
H18A	1.1866	−0.0209	0.1927	0.173*	0.57 (3)
H18B	1.0723	0.1140	0.1735	0.173*	0.57 (3)
H18C	1.0220	−0.0555	0.1609	0.173*	0.57 (3)
C17B	0.967 (2)	−0.045 (3)	0.2318 (8)	0.136 (9)	0.43 (3)
H17C	1.0491	0.0111	0.2524	0.164*	0.43 (3)
H17D	0.9406	−0.1378	0.2511	0.164*	0.43 (3)
C18B	1.027 (3)	−0.078 (5)	0.1915 (11)	0.201 (15)	0.43 (3)
H18D	1.1232	−0.1419	0.1977	0.302*	0.43 (3)
H18E	1.0608	0.0124	0.1729	0.302*	0.43 (3)
H18F	0.9469	−0.1334	0.1705	0.302*	0.43 (3)
N1	0.3493 (2)	−0.0030 (2)	0.31573 (6)	0.0420 (4)	
N2	0.4446 (2)	0.12399 (19)	0.30664 (6)	0.0407 (4)	
O1	0.02722 (17)	0.37131 (17)	0.44539 (5)	0.0441 (3)	
O2	0.5521 (2)	0.35450 (19)	0.32838 (6)	0.0604 (4)	
O3A	0.776 (2)	0.037 (5)	0.3148 (7)	0.069 (5)	0.50 (10)
O3B	0.756 (4)	−0.019 (7)	0.3125 (11)	0.088 (6)	0.50 (10)
O4	0.8114 (2)	0.0497 (3)	0.22828 (6)	0.0776 (7)	
Cl1	0.04000 (9)	0.85141 (8)	0.60423 (3)	0.0730 (2)	

### Atomic displacement parameters (Å^2^)

**Table:**

	*U*^11^	*U*^22^	*U*^33^	*U*^12^	*U*^13^	*U*^23^
C1	0.0478 (12)	0.0520 (13)	0.0493 (11)	−0.0015 (11)	0.0142 (10)	−0.0084 (11)
C2	0.0546 (14)	0.0482 (12)	0.0626 (13)	0.0075 (12)	0.0102 (11)	0.0050 (12)
C3	0.0488 (12)	0.0569 (13)	0.0443 (10)	0.0067 (11)	0.0026 (9)	0.0085 (11)
C4	0.0382 (10)	0.0484 (11)	0.0358 (9)	−0.0006 (9)	0.0078 (8)	−0.0005 (9)
C5	0.0345 (10)	0.0498 (12)	0.0359 (9)	0.0013 (8)	0.0070 (8)	0.0022 (10)
C6	0.0447 (12)	0.0665 (15)	0.0367 (10)	−0.0003 (10)	0.0053 (9)	−0.0045 (11)
C7	0.0416 (11)	0.0528 (13)	0.0401 (10)	0.0069 (9)	0.0050 (9)	0.0063 (10)
C8	0.0390 (11)	0.0473 (12)	0.0430 (10)	0.0028 (9)	0.0124 (9)	0.0046 (10)
C9	0.0465 (12)	0.0468 (12)	0.0483 (11)	−0.0017 (10)	0.0151 (9)	−0.0004 (11)
C10	0.0324 (9)	0.0403 (11)	0.0377 (9)	0.0021 (8)	0.0025 (7)	−0.0010 (9)
C11	0.0407 (11)	0.0402 (11)	0.0433 (10)	−0.0007 (9)	0.0069 (8)	−0.0071 (9)
C12	0.0364 (10)	0.0396 (10)	0.0466 (10)	−0.0023 (9)	0.0049 (8)	−0.0003 (9)
C13	0.0342 (9)	0.0410 (11)	0.0400 (9)	−0.0008 (8)	−0.0001 (8)	−0.0031 (9)
C14	0.0514 (13)	0.0480 (13)	0.0549 (12)	−0.0116 (11)	0.0066 (10)	−0.0053 (12)
C15	0.0453 (11)	0.0537 (13)	0.0390 (10)	0.0022 (11)	0.0098 (9)	−0.0005 (10)
C16	0.0521 (13)	0.0725 (18)	0.0463 (12)	0.0110 (12)	0.0144 (11)	0.0093 (13)
C17A	0.061 (5)	0.144 (10)	0.064 (4)	0.038 (5)	−0.005 (4)	0.003 (6)
C18A	0.061 (5)	0.197 (12)	0.088 (6)	0.058 (6)	0.045 (4)	0.051 (8)
C17B	0.068 (7)	0.191 (18)	0.150 (13)	0.082 (9)	0.079 (8)	0.109 (12)
C18B	0.098 (14)	0.31 (4)	0.195 (19)	0.098 (18)	−0.032 (12)	−0.15 (2)
N1	0.0421 (9)	0.0420 (10)	0.0420 (9)	−0.0047 (7)	0.0043 (7)	−0.0046 (8)
N2	0.0385 (9)	0.0433 (9)	0.0403 (8)	−0.0028 (8)	0.0092 (7)	−0.0037 (8)
O1	0.0458 (8)	0.0520 (9)	0.0345 (6)	0.0025 (7)	0.0015 (6)	−0.0032 (7)
O2	0.0626 (10)	0.0545 (10)	0.0640 (9)	−0.0194 (9)	0.0233 (8)	−0.0068 (9)
O3A	0.063 (4)	0.096 (10)	0.047 (4)	0.021 (5)	0.006 (3)	0.022 (5)
O3B	0.086 (7)	0.110 (15)	0.069 (5)	0.038 (8)	0.029 (4)	0.046 (7)
O4	0.0519 (10)	0.1259 (19)	0.0549 (9)	0.0329 (11)	0.0193 (8)	0.0216 (11)
Cl1	0.0791 (5)	0.0674 (4)	0.0725 (4)	−0.0034 (4)	0.0133 (3)	−0.0271 (3)

### Geometric parameters (Å, º)

**Table:**

C1—C6	1.378 (3)	C13—C14	1.498 (3)
C1—C2	1.392 (3)	C14—H14A	0.9600
C1—Cl1	1.742 (2)	C14—H14B	0.9600
C2—C3	1.376 (3)	C14—H14C	0.9600
C2—H2	0.9300	C15—N2	1.461 (2)
C3—C4	1.380 (3)	C15—C16	1.504 (3)
C3—H3	0.9300	C15—H15A	0.9700
C4—O1	1.370 (2)	C15—H15B	0.9700
C4—C5	1.391 (3)	C16—O3A	1.182 (17)
C5—C6	1.401 (3)	C16—O3B	1.234 (17)
C5—C7	1.439 (3)	C16—O4	1.319 (2)
C6—H6	0.9300	C17A—C18A	1.316 (15)
C7—C8	1.345 (3)	C17A—O4	1.470 (12)
C7—H7	0.9300	C17A—H17A	0.9700
C8—O1	1.380 (3)	C17A—H17B	0.9700
C8—C9	1.488 (3)	C18A—H18A	0.9600
C9—C10	1.520 (3)	C18A—H18B	0.9600
C9—H9A	0.9700	C18A—H18C	0.9600
C9—H9B	0.9700	C17B—C18B	1.16 (3)
C10—C11	1.344 (3)	C17B—O4	1.493 (18)
C10—C13	1.434 (3)	C17B—H17C	0.9700
C11—C12	1.446 (3)	C17B—H17D	0.9700
C11—H11	0.9300	C18B—H18D	0.9600
C12—O2	1.228 (2)	C18B—H18E	0.9600
C12—N2	1.367 (3)	C18B—H18F	0.9600
C13—N1	1.305 (2)	N1—N2	1.365 (2)
			
C6—C1—C2	122.8 (2)	H14A—C14—H14C	109.5
C6—C1—Cl1	119.41 (17)	H14B—C14—H14C	109.5
C2—C1—Cl1	117.82 (18)	N2—C15—C16	111.14 (17)
C3—C2—C1	120.7 (2)	N2—C15—H15A	109.4
C3—C2—H2	119.7	C16—C15—H15A	109.4
C1—C2—H2	119.7	N2—C15—H15B	109.4
C2—C3—C4	116.43 (19)	C16—C15—H15B	109.4
C2—C3—H3	121.8	H15A—C15—H15B	108.0
C4—C3—H3	121.8	O3A—C16—O3B	24.8 (15)
O1—C4—C3	125.54 (18)	O3A—C16—O4	123.2 (9)
O1—C4—C5	110.30 (18)	O3B—C16—O4	123.6 (10)
C3—C4—C5	124.13 (19)	O3A—C16—C15	125.6 (9)
C4—C5—C6	118.7 (2)	O3B—C16—C15	124.9 (11)
C4—C5—C7	105.27 (18)	O4—C16—C15	109.41 (19)
C6—C5—C7	136.0 (2)	C18A—C17A—O4	115.8 (9)
C1—C6—C5	117.23 (19)	C18A—C17A—H17A	108.3
C1—C6—H6	121.4	O4—C17A—H17A	108.3
C5—C6—H6	121.4	C18A—C17A—H17B	108.3
C8—C7—C5	107.06 (19)	O4—C17A—H17B	108.3
C8—C7—H7	126.5	H17A—C17A—H17B	107.4
C5—C7—H7	126.5	C17A—C18A—H18A	109.5
C7—C8—O1	111.11 (19)	C17A—C18A—H18B	109.5
C7—C8—C9	134.0 (2)	H18A—C18A—H18B	109.5
O1—C8—C9	114.88 (17)	C17A—C18A—H18C	109.5
C8—C9—C10	114.63 (18)	H18A—C18A—H18C	109.5
C8—C9—H9A	108.6	H18B—C18A—H18C	109.5
C10—C9—H9A	108.6	C18B—C17B—O4	115.6 (19)
C8—C9—H9B	108.6	C18B—C17B—H17C	108.3
C10—C9—H9B	108.6	O4—C17B—H17C	108.4
H9A—C9—H9B	107.6	C18B—C17B—H17D	108.5
C11—C10—C13	118.60 (17)	O4—C17B—H17D	108.4
C11—C10—C9	123.06 (18)	H17C—C17B—H17D	107.4
C13—C10—C9	118.33 (18)	C17B—C18B—H18D	109.4
C10—C11—C12	121.13 (18)	C17B—C18B—H18E	109.6
C10—C11—H11	119.4	H18D—C18B—H18E	109.5
C12—C11—H11	119.4	C17B—C18B—H18F	109.4
O2—C12—N2	120.95 (17)	H18D—C18B—H18F	109.5
O2—C12—C11	124.88 (19)	H18E—C18B—H18F	109.5
N2—C12—C11	114.17 (17)	C13—N1—N2	117.70 (16)
N1—C13—C10	122.17 (19)	N1—N2—C12	126.10 (15)
N1—C13—C14	115.89 (18)	N1—N2—C15	114.57 (16)
C10—C13—C14	121.94 (17)	C12—N2—C15	119.25 (16)
C13—C14—H14A	109.5	C4—O1—C8	106.25 (15)
C13—C14—H14B	109.5	C16—O4—C17A	119.7 (5)
H14A—C14—H14B	109.5	C16—O4—C17B	114.9 (7)
C13—C14—H14C	109.5	C17A—O4—C17B	17.9 (15)

### Hydrogen-bond geometry (Å, º)

**Table:**

*D*—H···*A*	*D*—H	H···*A*	*D*···*A*	*D*—H···*A*
C6—H6···O3*B*^i^	0.93	2.37	3.291 (19)	170
C15—H15*B*···O2^ii^	0.97	2.34	3.278 (3)	161
C18*A*—H18*A*···O2^iii^	0.96	2.41	3.310 (10)	156

Symmetry codes: (i) *x*−1/2, −*y*+1/2, −*z*+1; (ii) −*x*+1, *y*−1/2, −*z*+1/2; (iii) −*x*+2, *y*−1/2, −*z*+1/2.
