# Supplementary figures and images for: Crystal structure of ethyl 2-{4-[(5-chloro-1-benzo­furan-2-yl)meth­yl]-3-methyl-6-oxo-1,6-di­hydro­pyridazin-1-yl}acetate
Source: Acta Crystallogr E Crystallogr Commun. 2015 Apr 9;71(Pt 5):o291–2. doi: 10.1107/S2056989015006301 (PMC4420114; doi:10.1107/S2056989015006301)

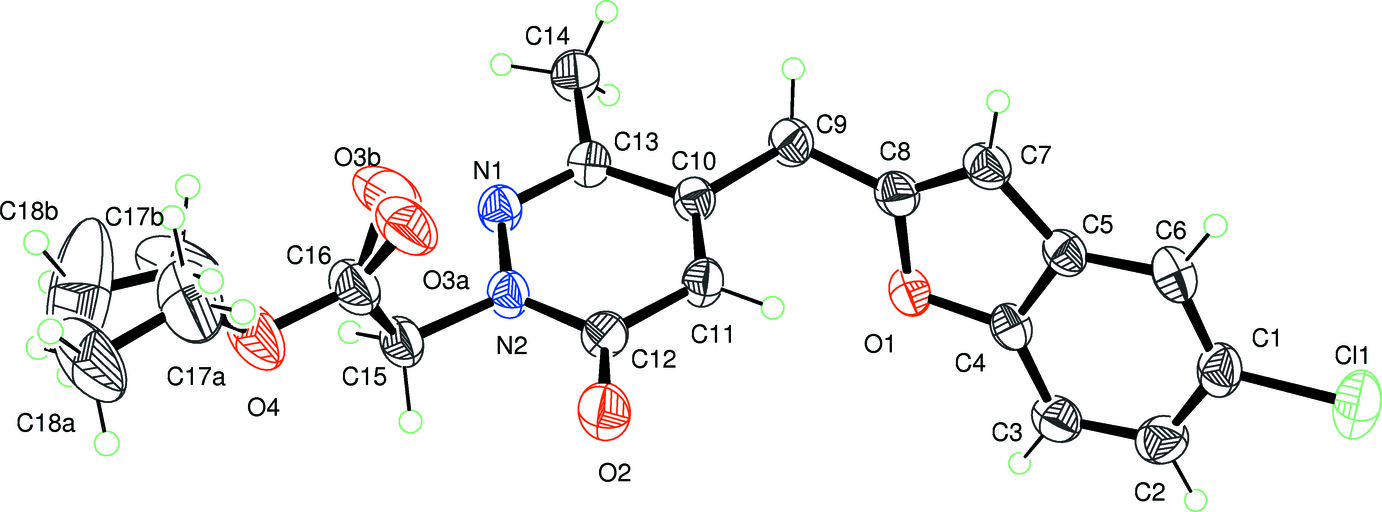

Supplement: Supplementary file 4 [file e-71-0o291-fig1.tif]

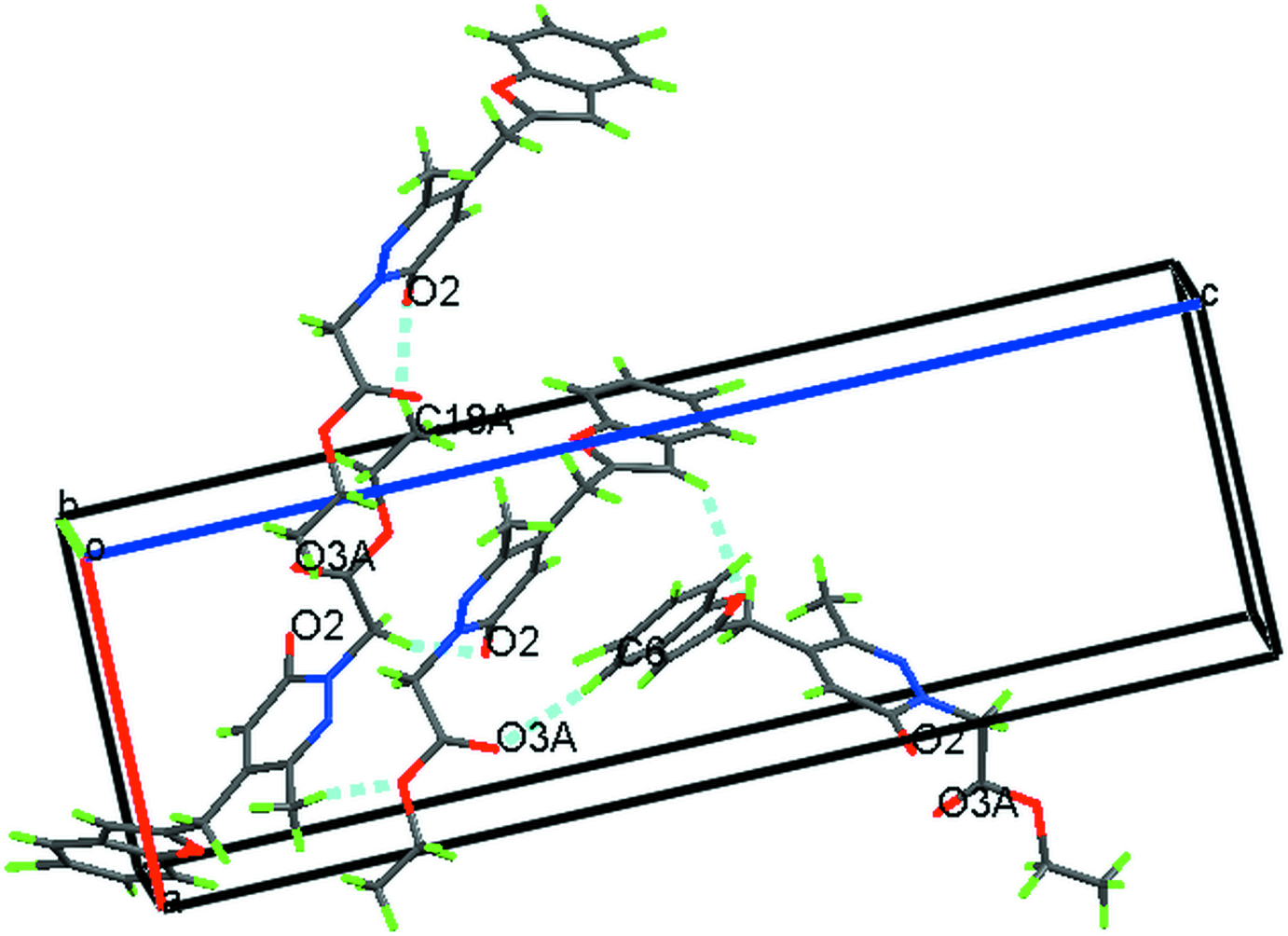

Supplement: Supplementary file 5 [file e-71-0o291-fig2.tif]
